# The Early Stages of Dental Caries

**Published:** 1917-06

**Authors:** Douglas E. Caush


### THE EARLY STAGES OF DENTAL CARIES

BY DOUGLAS E. CAUSH, L.D.S.

Teeth are immune, or otherwise, according to the amount of calcification that takes place at the vulnerable points at the time of their development. That the calcification of the teeth is not as perfect as our textbooks suggest, is the opinion I have formed after the examination of over a thousand sections of enamel from human teeth.

As Dr. Colyer in his December paper, published in December, 1916, *Dental Record,* deals with the earliest stages of caries at what he calls the "facets of contact," I shall confine my remarks, about the structure of the enamel and the action of the acids, to that portion of the tissue under consideration.

Immediately under Nasmyth's membrane it is not unusual to find some portion of the enamel more perfectly calcified than others and producing a more or less homogeneous appearance. In other portions of the tissue there are to be found in various quantities portions of uncalcified, or semicalcified tissue between the prisms, taking the form of lines passing towards the dentine; these spaces are to be found in numbers from that of a single line to bundles of considerable width, and in no two teeth are they the same. In most teeth portions of the margin will be comparatively free from them, and then a number may be seen grouped together. Differing in length, as well as in position, they are usually to be found in large numbers at the cervical margin, surrounding the sulci in the crown, and at those points where the teeth touch each other; *they are always to be found where deep caries is commencing.* That they differ in character from the normal cementing substance between the prisms is confirmed by the fact that when viewed by transmitted light (under the microscope) the appearance is quite different from normal spaces between the prisms. They are usually wider than the normal spaces of cementing substance, and are not to be found all over the section, as they would be, if formed of the usual cementing substance between the prisms. Their presence, or absence is, I believe, one of the principal causes why teeth are either susceptible to, or immune from caries at these points.

I may make my meaning clearer, and at the same time confirm the views expressed by Dr. Colyer, if a short description of what I believe takes place when acid formed from the soluble carbohydrates acts upon the enamel at these interdental spaces: (1) Upon a tooth where the semicalcification (previously described) exists; (2) upon a tooth when the calcification has been more perfect and there is, as a consequence, the absence of these markings.

1. In both cases the early or preliminary stage will be the same, the movement of the teeth against each other that produce the "facets of contact" with break down nature's first line of defense (namely, Nasmyth's membrane), as this must be destroyed by the friction which forms these facets at the point of contact. I do not think the acid developed from the carbohydrates can have any effect upon the membrane (as it will resist the action of strong acids for a considerable time) except that, when it is worn thin by the friction, the acid may work under the membrane, and by destroying the surface of the enamel produce a roughened surface that will assist in the final destruction of the membrane. The acids, doubtless, play an important part in the development of the facets by softening the surface of the enamel where the membrane is absent, and as soon as these facets are formed the first stage in the development of caries is reached. The further development is controlled by the condition of the enamel at, and near, the facets. If this tissue has been imperfectly calcified in the manner previously described, the ends of what I have called the semicalcified tissue will be exposed to the action of the acids. By the penetration of the acids into this uncalcified, or semicalcified tissue, and its action upon the sides of the prisms, small canals are formed, thus producing the early stages of caries, which we call white spots or white decay. When this semicalcified tissue is present in any quantity, or passes some distance (as it frequently does) into the enamel, the acid action continues to penetrate between the prisms of the enamel until the dentine is reached. Soon after the canals have commenced to form, I believe (from the examination of some of my slides) bacteria with the power of developing acids pass into these canals and help to carry on the work of destruction by the acids they develop, and are thus carried into the dentine.

2. If the enamel is more perfectly formed and there is an absence of the semiuncalcified tissue, the action of the acids upon the enamel which is exposed as a result of the formation of the facets and upon the surrounding tissue where it can pass under the edges of Nasmyth's membrane, is as follows: The formation of white caries is the same as in the former case, only with this difference, that it will spread laterally from the facets in the form of semilunar cavities, forming very slowly and producing but very shallow cavities, unless it comes in contact with some of the less calcified tissue, when it will commence to penetrate as in the former case. If the enamel is perfectly calcified the caries appears to cease, leaving nothing but a roughened surface at, and near the points of contact.

In a short communication it is impossible to enter more fully into details about this important subject; the above has been written simply to confirm Dr. Colyer's views as expressed in his most interesting paper.

To those who wish to further study this subject microscopically, may I suggest that, in examining slides under the microscope, oculars of high power be used (I always use one magnifying eighteen times) as in grinding down hard sections the surface of the tissue near the margin may possibly be altered somewhat by the grinding and with a high ocular and a comparatively low objective, a magnification of from 400 to 600 diameters is obtained with much greater depth of focus (so that the tissue under the surface of the section can be examined with ease) than can be obtained by using a low-power ocular, and high objectives.—*Dental Record,* March, 1917.

